# Applied Psychology

**Published:** 1915-06

**Authors:** Florence R. Ramer


### The Department of Eugenics, Mental and Social Hygiene

CONDUCTED BY
DR. MALONE DUGGAN
813-814 Gibbs Building, San Antonio, Texas
All communications for this department should be sent to DR. DUGGAN

### Applied Psychology.

BY FLORENCE R. RAMER.

Psychology to the average adult means "queer doin's." There has been, I suppose, more real opposition to the progress of this science, baffling enough by its very nature, than to any other in modern experience. Folks object to being analyzed. They confuse an interest in mental processes with an interference with the Deity. Psychology means pretty much the same to them as spiritualism and "conjurin'." It has been steady and uphill work, this fighting superstition. For without subjects there can be no analyses. We have finally educated ourselves out of our natural predisposition to ridicule things of which we know nothing, and psychology is coming into its own, not as a set of abstract Martian theorems, but a set of live, working principles.

Applied psychology—that branch of the entire subject that I have elected this evening to outline in a sketching way—is as absorbing a subject to the primarily initiated as electricity is to a boy, or poetry to a girl. Indeed, it has elements of both—the vital, compelling spark of the one, and the mystery and intangibility of the other. It evidences itself, I think, in five important branches of human endeavor: education, law, economics, medicine and culture.

Educational psychology is the most extensive and perhaps the most important phase. The old rudimentary method of instilling into the infant mind the basic principles of the three R's by rote, rattan and reward, contained no slightest trace of the educational psychology we have evolved today. If Tommie didn't say his multiplication tables in the exact way that Willie sang them, he was made to feel highly uncomfortable, which is a euphonious way of expressing it. Education was a hand-me-down garment made to fit each child, not by altering the garment, but by altering the child. His panties were cut to fit him, but he was beaten into the hairy shirt of algebra, whether he was large or small, strong or weak, disposed or no. Enough of the Dark Ages.

We are now embarking on an age of vocational training which, after all, is but the result of patient research along psychological lines. We now take into consideration the personal equation of the child, a hitherto unknown or ignored quantity. Can you imagine a gardener planting orchards on a lawn; or a farmer trying to raise cotton in Alaska; or a stock fancier feeding ice cream to his chickens; or a physician prescribing mince pie to a six months' old infant? And yet that is what we have been doing in the schools—planting the same seeds, in the same way, with the same culture, regardless of the soil.

The development of each child's latent and peculiar abilities to the furtherance of the child's usefulness as a citizen, and of his ultimate happiness and success, is the work of the educational psychologists. We no longer attempt to make a dreamy, poetical, impractical child swallow willy-nilly the large and, to him, nauseous doses of mathematics. We no longer torture the vigorous, healthy, normal boy with a continuous diet of what seems to him to be insipid, vacuous, irrelevant poetry. He wants life, and the work of his hands to divert the tremendous amount of vitality that seeks outlet in mischief.

We have discovered that the child who seems not only uninterested in the ordinary routine of the school room, but who is actually dull, show marked talents in other directions and a feverish energy when the things he likes to do are made the subjects of his lessons. Some need their food predigested; some need to do their own chewing; some need tactful treatment. Some need pushing; some need holding back, and others need, not the switch, but the doctor. A cross, disobedient, sluggish pupil is nine out of ten times a more or less sick child. And while I realize that this is but a string of platitudes to you, the point I wish to make is this: the enormous strides that have been made in educational fields are due in the main to the results of psychology's invasion of that domain. Common sense, after all the rarest of attributes, is the keynote in the teaching of the young.

The psychological aim of education is to develop the mental self, and this growth can come only through activity. Curiosity, invention, the esthetic demands, convention, morality, emulation, competition, the sense of ownership, the instinct of self-preservation, the constructive impulse are some of the familiar ways in which the immature mind develops itself through its reactive powers. In proportion as we place within reach of this mental hunger the food it craves in an appetizing form, just so will we have the corresponding development and growth.

To play on the three leading determining factors in the mental life of a child; namely, interest, imitation and effort, and to apply this psychological knowledge for his benefit, represents the educational ideal. Right here let me add that the teacher in the schools has much more opportunity to influence the growth of the pupil than even the mother has. A child after he once starts going to school is under the direct supervision of his mother largely only when he eats and sleeps. In the face of such a condition, to place in front of a class of eager, joyous little animals who are radiant with health and vitality, a weazened, dried, mechanical, unprepossessing, dowdily-dressed, sexless female, or an effeminate male for five or six hours a day, is nothing short of a punishment. Whether they realize it or no, they are impressed with the personality of the teacher. As long as we give a teacher twice as many pupils as she ought to have, with one-half the requisite amount of time in which to teach them, underpay and underfeed her, just so long will we have these flat tasteless, non-entities as the instructors of our young.

Now I am going to talk shop. The legal aspect of applied psychology is highly interesting to me. If I could learn the psychology of the court room and practice it, I should, gentlemen, be a *real* lawyer instead of a rather dubious imitation. To establish a sound revelation between himself and his client, represents one of the most serious problems that confront the young lawyer. The lack of candor, the desire to shield himself from the consequences of his folly or wrongdoing are but a few of the average client's pleasant little subterfuges. The lawyer must, first of all, put himself and his client on a basis of absolute candor—that is, the client must be candid with him. Let me be understood: there is a type, a prevalent type, that, as in the practice of medicine, must be kept in ignorance if you would have him respect your advice. To destroy the illusion of some God-given faculty in the lawyer that makes him know more is sometimes to defeat one's own ends.

Cross-examination is one of the most fertile fields for the psychology student. Upon the will-strength of the witness and the shrewdness of the examiner depends the straight or the contradictory testimony. Sugggesting to one's own witness what one wants him to answer is a procedure that we all must follow at times. The other day I had to procure a witness at the last moment and had no time to interview her before the case was called. I put her on the stand, and, after a few preliminaries, asked her a question that contained its own answer, and upon which hung the weight of her testimony. Counsel for the defense objected, and the court ruled with him—justly so—on the ground that it was a leading question. I worded it differently and got the information I was looking for, for I had conveyed to her just what I was driving at.

The emotional appeal to the jury, a powerful weapon in the hands of the expert, and a boomerang in the hands of the amateur, the effect upon them of a stack of law books, whether one uses them or not, the employment of sarcasm, irony, and ridicule directed against one's opponent to make him lose his temper and control, the psychological effect of poise on the court, and going on a bare assumption to surprise an admission, all are but a few of the tricks of the trade. They have been used, I suppose, since time immemorial, but it is only in recent times that they have become the conscious instruments of the barrister.

Let me close this branch of my subject with an incident showing how suggestion can be employed to the undoing of a witness. In a personal injury suit, the plaintiff himself took the stand. His counsel in emotional tones brought out the story of his injury, and had him raise his arm to show the jury how it had been crippled. His hand went to his shoulder—no higher.

On cross-examination, counsel for the defense, in a soothing, sympathetic voice, asked the witness to show *him* how high he could raise his arm. Witness did so, with much pain and effort. The lawyer then said quickly, "Now show the gentlemen of the jury how high you could raise you arm *before* you were hurt." Before he could realize what he was doing, up it shot, full-length.

Physicians are using, as civilization advances, less medicine and more mentality on their patients. Suggestion, confidence, optimism, and, in a lesser degree, fear, are the modern doctor's available and high efficacious resources. Hypnotism and intensified suggestion, have been used by them to advantage in nervous maladies, dipsomania, mild obsessions, and the like. Some quite remarkable cures have been affected along these lines, and the number of cases in which the will-power of the subject has been increased through this means, shows how fallacious is the popular belief that hypnosis is largely the overpowering of a weak will by a strong one.

The confidence that one feels in the family physician, and the indubitable sense of relief that follows his visit, is the direct result of his suggestion to you, and is largely curative, as he knows. He also knows when to give and when to withhold information as to the patient's condition, according to the effect he wishes to produce. He knows when to enforce obedience to his orders through fear, or by appealing to reason. Some people need to be told the very worst regarding their maladies at the outset. With others this course might be fatal. The successful practitioner is he who analyzes swiftly and surely his patient's mentality, and acts accordingly. This insight is, in some cases, as important as the physical diagnosis.

The doctor has the widest philanthropic scope of any professional; his subject is suffering; his object, relief. His greatest asset is the confidence he inspires, and the laying-on of his hands cures at times where a drug might fail. I fear to go into this subject any deeper, lest I be treading where angels are too wise to go. Suffice it that my point is this: the mental attitude of the doctor can and does influence his patient to a remarkable degree.

To descend to prosaic economics after the poesy of high philanthropy is a cold plunge. And yet the merchant, too, has his own psychology to apply. The successful business man is the scientific one, whether he be analytical by nature or through effort. Advertising represents the field in which the applied psychology of economics has the widest scope.

Any one with a modicum of brains can handle business that is thrust upon him. But to make for one's products a new market; to create a demand to fit the supply; to excite envy, the sense of economy, the desire to appear as well as one's neighbor, the love of home, children and luxury; to impress on a fickle public the desirability of one's wares and to reach them through one or more of these appeals: this is the purpose of forceful and successful advertising.

It must go through four separate and distinct phases of the emotions; namely, attention, interest, desire, and the resolution to buy. The scientifically constructed advertisement follows this psychological process in its logical order. First of all, a display must attract attention. It might have the merits of all possible benefits, and, if it does not challenge the reader, it is wasted. Color, form, eccentricity, pictures, all have their place and uses in attracting the eye.

Next, one must arouse interest. The story must be told in a concise, direct, simple way. No fine language, no emotional flights, no hyperbole, just a plain statement of fact with the purchaser's needs and mental attitude *only* in mind. It isn't what I want to sell but what he wants to buy that must be the burden of my story. After having gained his attention, and aroused his interest, the next thing for me to do is to create a desire through an appeal to the average man's or woman's most frequent emotions—those enumerated above as the leading motives for purchase. An advertisement must be eminently truthful to be successful. No overstatement will bring permanent results, and the value of understatement is just beginning to be appreciated. To my mind, the strongest advertisement that I ever saw was in an English magazine, and bore the clear cut of a very handsome diamond wrist-watch. Below in small type was the merchant's name and address, and in moderate sized type ran the legend, "Quite reliable—$225."

A merchant's reliability, his honor, his fair dealing, are all his psychological assets. They may form and often do form the determining factors in inducing a purchase, especially where competition is keen and offerings nearly identical. The catchy phrase, the insidious suggestion, or the forcibility of an argument swing business his way, even when other things are equal. The wise merchant selects his employes with care. They add to, or detract, from the tone of his establishment, and create a definite impression, recognized or otherwise, on the would-be purchaser. One had rather pay a bit more and be waited on by a gentle-voiced, calm, unassuming, accommodating sales girl, than to pay a bit less and confront a bejeweled, gum-chewing, whitewashed confection who "Dearies" you out of the store.

It seems a paradox to speak of the practical side of cultural psychology; but is it?

The minister is a most devout disciple of applied psychology. He makes his appeal with malice aforethought, as it were, and the successful divine is he who uses these tools skilfully. A minister's dignity, his personal appearance, his voice, his gestures, are all part and parcel of the religion his flock imbibes. A preacher with theatrical mannerisms cannot seem sincere, however much so he may be. A comfortably-fed, rotund clergyman does not make a deep impression if he preach aceticism. What he must do is to pattern his preachings appropriately; in other words, the minister whose church is filled on Sunday is he who makes the most of *himself*. Exaggerated dignity does not become some; neither does levity become others. The Catholic Church and its services fasten on the senses and through them appeal to the religious emotion. The Oriental atmosphere of mystery, the heavy odors of incense, the rich garments of the priests, the Latin chanting, the dim lights, the sombre statues, and the rigid ceremonials carry the worshipper into a semi-ecstatic state without his knowledge or volition. They all make a distinctly sensory appeal, and religion is but a sort of sublimated sensuousness.

Painting, sculpture, and music are of indisputably practical value. The inspiration one derives when confronted with a work of art, even though it be but a fleeting impression, is of height, beauty and purity, and the medium of canvas, marble or musical instrument leaves us psychologically just that much richer.

Commercialism has invaded all three, and while we may and probably do deplore the uses to which they are at times diverted, there is a psychological basis for almost every sporadic adaptation of art, so-called. The modern ragtime is an insidious call to the barbaric love of syncopated rhythm. In almost every savage tribe you can find what the modern American would call a "rag." The huge success of the popular song-writers is founded on the psychology of the hyper-civilized individual, living in artificial surroundings, impelled by an artfully construed rhythm to revert to savagery. Ragtime, to my mind, is not a prostitution of music. I love my Bach and Beethoven and my Wagner quite well, but the irresistible appeal of the seemingly senseless rag is in itself its own justification. To abandon it causes one to feel, and the subsequent heightening of tone is enough excuse for being.

Higher education for women is another phase of modern evolution that has a fertile basis for exploration from this present point of view. It is fortunately no longer necessary in a body of even semi-intelligent individuals to seek to convert them to the cause of the emancipation of women. If you gentlemen think that I am going to embrace this golden opportunity to harangue you on the subject of woman's suffrage, you are mistaken. I pay you the subtle compliment of believing that you are all enthusiastic supporters of the movement, and will take for granted your toleration if not your active co-operation.

To sum up: in every walk of our prosaic and hideously complex life we find the romance of reason and psychology. Success is but the manifestation of a keen insight into the needs of one's particular audience—and supplying them. Education, law, medicine, commerce, and culture have each their own tricks and trade secrets, and so far from true is it that tricks are despicable and unworthy that I will say this: he trades best who thinks deepest. In other words, it is a wise seller that knows his buyer.

There is a splendid chance to rhapsodize on the "why's" of psychology, and to moralize on the immense influence for both good and evil its tenets may have in the hands of an earnest student. That, however, is neither my province nor my intention. I am content to state that to seek for the underlying causative forces for seeming irrelevant effects, to hitch them up, as it were, is as absorbing a study as the batting average of Ty Cobb, or the isolation of some elusive bacteria.

**Read before the San Antonio Scientific Society April 30, 1915.**

